# The Impact of Clinical and Morphometric Parameters on Hematopoietic Engraftment Following High-Dose Chemotherapy and Autologous Stem Cell Transplantation in Germ Cell Tumors

**DOI:** 10.3390/medicina61091655

**Published:** 2025-09-11

**Authors:** Ömer Faruk Kuzu, Nuri Karadurmuş, Ahmet Gazi Acar, Nebi Batuhan Kanat, Çağlar Köseoğlu, Ayşegül Dumludağ, Alper Topal, Doğan Bayram, Efe Cem Erdat, Musa Barış Aykan, Uğur Bozlar, İsmail Ertürk

**Affiliations:** 1 Cankiri State Hospital, 18100 Çankiri, Turkey; 2 Gulhane Research & Training Hospital, 06010 Ankara, Turkey; drnkaradurmus@yahoo.com (N.K.); nebibatuhan.kanat@sbu.edu.tr (N.B.K.); drdoganb@gmail.com (D.B.); cemerdat@gmail.com (E.C.E.); musabarisaykan@gmail.com (M.B.A.); ubozlar@yahoo.com (U.B.); ierturk@hotmail.com (İ.E.); 3 Sincan Training and Research Hospital, 06930 Ankara, Turkey; g.acar353@gmail.com; 4 Gaziantep City Hospital Gaziantep, 27470 Şahinbey, Turkey; drcaglarkoseoglu@hotmail.com; 5 Erzurum Regional Education and Research Hospital, 25240 Erzurum, Turkey; dr.aysegulcomakli@gmail.com; 6 School of Health, Tokat Gaziosmanpasa University, 60250 Tokat, Turkey; dralpertopal@gmail.com

**Keywords:** germ cell tumor, high-dose chemotherapy, autologous stem cell transplantation, body composition, visceral adiposity, engraftment

## Abstract

*Background and Objectives*: Relapsed or refractory germ cell tumors are commonly treated with HDCT/ASCT, but robust predictors of hematopoietic recovery are limited. Quantitative CT-based metrics of body composition are readily available, but their prognostic value for post-transplant engraftment remains uncertain. We investigated whether muscle and fat indices derived from routine CT scans are associated with the pace of hematologic recovery after HDCT/ASCT. *Materials and Methods*: This retrospective study analyzed a single-center cohort (*n* = 43) with relapsed/refractory GCT undergoing HDCT/ASCT. CT within 6 months pre-HDCT/ASCT was analyzed at L3 to derive the Skeletal muscle index, Psoas muscle index, Subcutaneous fat area, Visceral fat area, Total fat area, Visceral-to-subcutaneous fat area ratio. Primary endpoint: The engraftment time post-ASCT. Spearman’s ρ was used for univariable associations; multivariable linear regressions were adjusted for age, Hb, weight, and BSA to evaluate the independent effects. The significance was set at *p* < 0.05. *Results*: The median hematologic engraftment duration was 12.0 days, and the engraftment duration was positively correlated with age and negatively with hemoglobin. According to the multivariable analysis, older age and lower hemoglobin independently predicted longer engraftment; body weight and BSA were not significant. Among the morphometrics, only the VFA/SFA ratio was associated with delayed engraftment. The SMI, PMI, and TFA were not significant. As expected, after HDCT, grade 4 neutropenia and thrombocytopenia occurred in all patients. *Conclusions*: In relapsed/refractory GCT treated with HDCT/ASCT, older age and lower post-transplant hemoglobin independently predicted a prolonged engraftment. Beyond traditional muscle/fat areas, a higher VFA/SFA ratio—reflecting visceral adiposity—is also associated with delayed recovery, suggesting that fat distribution may influence hematopoietic regeneration. These variables may support pre-transplant risk stratification and individualized supportive care.

## 1. Introduction

Germ cell tumors (GCTs) are curable, even in the setting of relapsed or refractory disease, but their treatment remains challenging [1]. Approximately one-quarter of patients initially treated with cisplatin-based combination regimens will experience disease relapse or exhibit refractoriness, necessitating alternative therapeutic strategies [2]. Despite this, durable remission or even cure can often be achieved through salvage treatments. The current therapeutic options for second-line treatment and beyond include conventional-dose chemotherapy (CDCT) and high-dose chemotherapy (HDCT), with both approaches widely utilized in clinical practice. Existing treatment guidelines indicate no definitive difference in efficacy between CDCT and HDCT [3]. HDCT, when administered in conjunction with peripheral autologous stem cell transplantation (ASCT), has been demonstrated to improve recovery outcomes in salvage settings [3]. Nonetheless, HDCT should be conducted in high-volume, experienced centers, presenting a significant limitation to its widespread implementation [4]. Given the rarity of relapsed or refractory GCTs, conducting phase III randomized clinical trials remains a challenge; thus, treatment decisions are frequently guided by retrospective evidence. Notably, most phase III trials evaluating HDCT have not demonstrated a significant survival benefit [3].

Emerging evidence indicates that body composition parameters—specifically skeletal muscle mass and adipose tissue distribution—are associated with treatment-related toxicities and the overall mortality in patients with cancer [5].

These parameters are commonly evaluated using diagnostic imaging modalities, including computed tomography (CT), dual-energy X-ray absorptiometry, magnetic resonance imaging, and bioelectrical impedance analysis. Among these, CT imaging has become the predominant modality in oncology-related body composition research, primarily because cancer patients frequently undergo CT scans as part of routine care [6,7,8,9]. Most studies utilize axial CT images at the third lumbar vertebra (L3) level due to its consistent visualization of multiple muscle groups and visceral fat compartments, as well as its widespread availability in abdominal imaging. However, alternative anatomical landmarks such as the psoas muscle, temporalis muscle, and thoracic vertebral levels have also been explored in recent literature for body composition analysis [10,11,12,13]. CT-derived body composition analysis provides valuable quantitative metrics, including the skeletal muscle area (used to define sarcopenia), intramuscular fat infiltration (indicative of myosteatosis), subcutaneous and visceral adiposity, and fat density. To date, most oncology studies have focused on the clinical relevance of sarcopenia and myosteatosis. For example, a recent study involving 78 lymphoma patients undergoing ASCT demonstrated that those with sarcopenia exhibited significantly poorer progression-free survival, although the adiposity-related parameters were not evaluated in that cohort [14]. Recently, research interest has expanded toward the prognostic value of adipose tissue metrics. Notably, some studies have reported paradoxical findings, such as an improved overall survival among patients with hematologic malignancies who exhibit higher levels of visceral adiposity [15,16].

In adults with lymphoma undergoing ASCT, sarcopenic obesity—defined by the coexistence of low muscle mass and an elevated body mass index (BMI > 25 kg/m^2^)—has been associated with an increased incidence of early post-transplant complications, including prolonged hospitalization, intensive care unit admission, and 30-day unplanned readmissions. Similarly, in multiple myeloma patients undergoing an ASCT, a reduced pre-transplant high-density muscle mass (≤80%) was linked to a higher risk of cardiovascular toxicity within the first 100 days post-transplant [17,18].

Achieving a successful ASCT requires both effective eradication of the underlying malignancy and complete bone marrow engraftment. Timely hematologic recovery is particularly crucial, as delayed engraftment may lead to increased early transplant-related complications and higher healthcare costs. In this context, identifying clinical or morphological factors that influence engraftment kinetics—such as body composition and laboratory parameters—may provide valuable insights for optimizing outcomes in patients undergoing HDCT/ASCT [19].

In addition to body composition metrics, we aimed to investigate the potential impact of other factors, such as post-transplant blood parameters (e.g., hemoglobin level) and patient age, on the kinetics of hematologic recovery following autologous transplantation.

We aimed to determine whether pre-transplant CT-derived body composition indices at L3 (SMI/PMI, TAMA, SFA, VFA, and VFA/SFA) and readily available clinical variables (age and pre-transplant hemoglobin) are associated with hematologic engraftment kinetics following HDCT/ASCT in relapsed/refractory GCT.

## 2. Materials and Methods

This retrospective study included 43 patients who were diagnosed with relapsed or refractory germ cell tumors, who underwent HDCT followed by ASCT at our institution. The demographic data, disease characteristics, treatment history, and clinical outcomes were extracted from electronic medical records.

### 2.1. CT-Based Body Composition Analysis and Image-Processing Protocol

**Figure 1:**
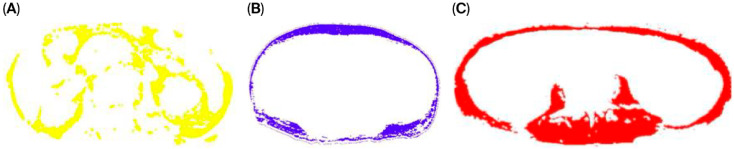
SFA mask (blue): subcutaneous adipose tissue external to the abdominal wall (**A**). VFA mask (yellow): intra-abdominal/visceral adipose tissue enclosed by the abdominal wall (**B**). TAMA mask (red): total abdominal skeletal muscle, including psoas, paraspinal, and abdominal wall muscles (**C**).

Body composition analysis was conducted using non-contrast abdominopelvic CT scans obtained from two different multi-detector CT systems: the Toshiba Aquilion One 320 and Toshiba Aquilion 64 (Otawara, Japan). The scans were acquired with standardized technical parameters, including a tube voltage of 100–140 kVp, tube current of 200–500 mA, rotation time of 0.4 s, and field of view of 400 mm. The CT images were retrieved in Digital Imaging and Communications in Medicine format via the institutional Picture Archiving and Communication System. The muscle and adipose tissue areas were quantitatively analyzed by an experienced radiologist using the CoreSlicer 1.0 software package. Measurements were obtained at the level of the L3, which was anatomically verified using coronal and sagittal multiplanar reformatted images. A predefined attenuation range of −30 to +130 Hounsfield Units was applied to segment the skeletal muscle tissue. The anterior abdominal wall muscles and bilateral psoas muscles were manually outlined using the software’s cursor tool. Following initial segmentation, the software automatically calculated the tissue areas. Any discrepancies were manually corrected using a brush tool to ensure precision (Figure 1).

### 2.2. Assessment of Body Composition Parameters and Index Calculations

The body composition parameters—including the subcutaneous fat area (SFA), visceral fat area (VFA), total muscle area (TAMA), and left and right psoas muscle areas—were evaluated using CT images obtained within 6 months before the HDCT/ASCT procedure. The images were acquired either from standard abdominal CT scans or from the CT component of the PET-CT examinations conducted within this period. Notably, TAMA also refers to the skeletal muscle area, as it represents the total cross-sectional area of the muscles at the level of the third lumbar vertebra (L3). Skeletal muscle measurements were obtained at the level of L3 using standardized imaging analysis techniques.

The skeletal muscle index (SMI) was calculated by dividing the cross-sectional area of skeletal muscle at the L3 level by the square of the patient’s height (cm^2^/m^2^):

SMI = [skeletal muscle area at L3]/height^2^

The psoas muscle index (PMI) was calculated similarly by dividing the sum of the bilateral psoas muscle areas at the L3 level by the square of the patient’s height (cm^2^/m^2^):

PMI = [sum of bilateral psoas muscle areas at L3]/height^2^

The total fat area (TFA) was defined as the sum of the visceral and subcutaneous fat areas (TFA = VFA + SFA). In addition, the VFA/SFA ratio was calculated to evaluate the distribution pattern of abdominal fat. The body surface area (BSA) was computed using height and weight data recorded prior to HDCT.

### 2.3. Post-HDCT Laboratory Assessment and Engraftment Criteria

Laboratory data were obtained during the post-HDCT period. Specifically, the analysis used the lowest recorded values of hemoglobin, the platelet count, the neutrophil count, sodium, calcium, and albumin, and the highest recorded values of creatinine, alanine aminotransferase (ALT), and aspartate aminotransferase (AST) during the transplant-related follow-up. These values were selected to reflect the peak hematologic and metabolic disturbances experienced during the engraftment phase.

Autologous stem cells (at least 2.5 million CD34+ cells per kilogram of body weight) harvested pre-HDCT were reinfused into patients two days after the last chemotherapy. GCSF therapy was then started the following day. All patients were administered antibiotics (levofloxacin), antivirals (valacyclovir), and antifungals (oral fluconazole) as a preventative measure against infections. In addition, prophylactic antiemetics were administered to manage nausea and vomiting, and oral care products were regularly included in the treatment protocol to address mucositis. Patients underwent daily monitoring of blood counts, biochemistry panels, C-reactive protein (CRP), and procalcitonin levels until engraftment. Platelet and erythrocyte transfusions were administered as needed to maintain platelet counts above 10,000/mm^3^ and hemoglobin levels above 8 g/dL, respectively.

In our cohort, the salvage chemotherapy was predominantly carboplatin–etoposide (CE); only a small subset received ifosfamide–carboplatin–etoposide (ICE), and no other salvage regimens were used.

Platelet engraftment was defined as achieving and maintaining a platelet count > 20,000/mm^3^ for at least three consecutive days without platelet transfusions, and neutrophil engraftment as an absolute neutrophil count ≥ 2000/mm^3^ maintained for at least three consecutive days without G-CSF. Engraftment was considered achieved when the predefined threshold for either neutrophils or platelets was met. Platelet and erythrocyte transfusions were administered as needed to maintain platelet counts above 10,000/mm^3^ and hemoglobin levels above 8 g/dL, respectively.

### 2.4. Statistical Analysis

Statistical analysis was performed using IBM SPSS Statistics Version 27.0. pearman’s rank correlation coefficient was used to assess the associations between variables, as the data did not follow a normal distribution and included ordinal or non-parametric measurements, and the sample size was relatively small. Multiple linear regression analysis was conducted to identify independent predictors of engraftment duration. A *p*-value < 0.05 was considered statistically significant.

## 3. Results

### 3.1. Patient Characteristics

A total of 43 patients with primary gonadal germ cell tumors who underwent HDCT/ASCT were included in this study, with a median age of 29 years. The majority (93.0%) had non-seminomatous histology, with mixed germ cell tumors being the most common subtype (74.4%). Pure seminoma was observed in 7.0% of cases. At initial diagnosis, the distribution of disease stage was balanced, with 48.8% of patients presenting with stage < 3, and 51.2% with stage ≥ 3 disease. According to the IGCCCG risk classification, 58.1% of the patients were categorized as poor risk, while 27.9% and 14.0% were classified as good risk and intermediate risk, respectively. The metastatic involvement at the time of diagnosis included the lungs in 60.5% of patients, the liver in 20.9%, bone in 16.3%, and the brain in 7.0%. Lymph node involvement was nearly universal, affecting 97.7% of the cohort. Regarding the response to therapy prior to HDCT/ASCT, 55.8% of patients achieved a complete response (CR) or partial response with negative tumor markers, while 41.9% showed a partial response (PR) with positive markers or stable disease (SD). Only one patient (2.3%) demonstrated progressive disease (PD). HDCT/ASCT was administered as consolidation after two lines of chemotherapy in 79.1% of patients and after three lines in 20.9%. The most commonly used HDCT regimen was CE (carboplatin and etoposide), which was received by 93.0% of patients; ICE (ifosfamide + carboplatin + etoposide) was used in the remaining 7.0%. Following HDCT/ASCT, 65.1% of patients achieved a CR or marker-negative PR. However, 20.9% experienced stable disease or marker-positive PR, and 14.0% had progressive disease, indicating a subset of patients with treatment-resistant disease despite intensive therapy (Table 1).

In the present study, the median hematologic engraftment duration was 12.0 days, the mean was 13.67 ± 3.60 days, and the range was 9 to 25 days. The 25th and 75th percentiles were 11.0 and 16.0 days, respectively.

### 3.2. Correlations Between Engraftment Duration and Pre-Transplant Morphological Parameters

**Figure 2:**
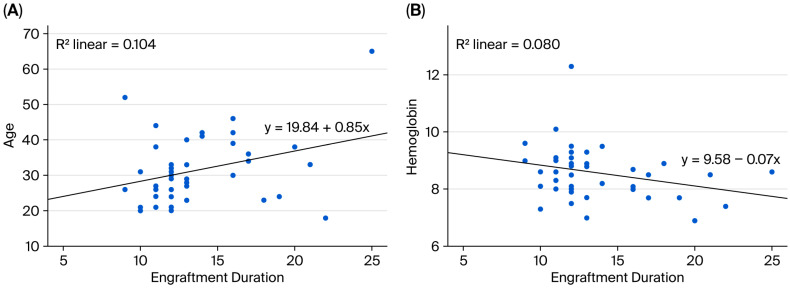
Relationship between engraftment duration and (**A**) age and (**B**) hemoglobin levels.

**Figure 3:**
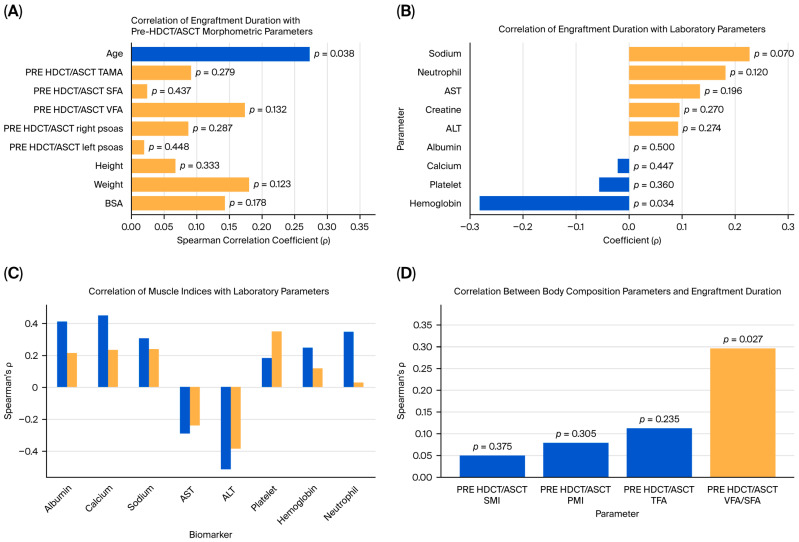
Analyses of engraftment duration’s correlations with morphometric, laboratory, and body composition parameters. (**A**) correlation of engraftment duration with pre-HDCT/ASCT morphometric parametres, (**B**) correlation engraftman duration an laboratory parameters, (**C**) correlations between muscle mass indices and laboratory parameters, (**D**) correlation between body composition parameters and engrafment duration.

Spearman’s rank correlation analysis was performed to examine the association between engraftment duration and pre-HDCT/ASCT anthropometric and body composition metrics. A statistically significant positive correlation between engraftment duration and age was observed (ρ = 0.274, *p* = 0.038). No statistically significant associations between the engraftment duration and pre-transplant area TAMA, SFA, VFA, or psoas muscle measurements were identified. However, weak positive but non-significant correlations with body weight (ρ = 0.181, *p* = 0.123) and BSA (ρ = 0.144, *p* = 0.178) were observed (Figure 2A and Figure 3A).

### 3.3. Correlations Between Engraftment Duration and Laboratory Parameters

Spearman’s correlation analysis was also conducted to assess the relationships between engraftment duration and various biochemical and hematologic markers. A significant negative correlation between the hemoglobin level and engraftment duration was identified (ρ = −0.281, *p* = 0.034), indicating that lower baseline hemoglobin levels may be associated with delayed hematopoietic recovery. Other laboratory markers, including the neutrophil count (ρ = 0.183, *p* = 0.120) and serum sodium levels (ρ = 0.229, *p* = 0.070), exhibited weak positive correlations, but these were not statistically significant. No meaningful correlations between engraftment duration and creatinine, AST, ALT, calcium, albumin, or platelet count were observed (Figure 2B and Figure 3B).

### 3.4. Multiple Linear Regression Analysis of Engraftment Duration

A multiple linear regression analysis was conducted to identify predictors of engraftment duration. The dependent variable was engraftment duration, with age, hemoglobin level, body weight, and BSA included as independent variables. The model approached statistical significance (F(4, 38) = 2.588, *p* = 0.052), explaining approximately 21.4% of the variance in engraftment duration (R^2^ = 0.214), with an adjusted R^2^ of 0.131. Two variables were found to be statistically significant predictors of engraftment duration. Age was positively associated with engraftment duration (B = 0.136, *p* = 0.018), whereas hemoglobin levels were negatively associated with engraftment duration (B = −1.256, *p* = 0.036). Body weight and BSA were not statistically significant predictors (*p* = 0.866 and *p* = 0.907, respectively). The residual analysis indicated a standard error of ±3.19 days, reflecting a moderate prediction error.

### 3.5. Correlations Between Muscle Mass Indices and Laboratory Parameters

The associations between the pre-HDCT/ASCT SMI and PMI and selected laboratory parameters were explored. The SMI showed significant positive correlations with albumin (ρ = 0.414, *p* = 0.003), calcium (ρ = 0.456, *p* = 0.001), and the neutrophil count (ρ = 0.350, *p* = 0.011). Additionally, it was significantly negatively correlated with ALT (ρ = −0.516, *p* < 0.001) and AST (ρ = −0.295, *p* = 0.028). The PMI was positively correlated with the platelet count (ρ = 0.353, *p* = 0.010) (Figure 3C, Table 2).

### 3.6. Correlation of Pre-HDCT/ASCT Body Composition Indices with Engraftment Duration

In this study, the associations between the pre-HDCT/ASCT body composition parameters and engraftment duration were evaluated. Among the variables analyzed, only the VFA/SFA ratio exhibited a statistically significant correlation with engraftment duration (Spearman’s ρ = 0.297, *p* = 0.027). No significant associations were observed between the SMI, PMI, or TFA and engraftment duration (*p* > 0.05) (Figure 3D, Table 3).

### 3.7. Treatment-Related Hematologic and Biochemical Toxicities

Severe hematologic toxicities were universally observed among patients. All individuals (100%) experienced grade 4 neutropenia and thrombocytopenia, reflecting the expected profound myelosuppression following HDCT. Anemia was also prominent, with the majority of patients exhibiting grade 2 anemia (69.8%), and a small subset developing grade 3 anemia (7.0%).

Regarding biochemical toxicities, a mild-to-moderate creatinine elevation was detected in 27.9% of patients, although grade 3 toxicity was uncommon (4.7%), and no grade 4 renal toxicity was recorded. Hepatic enzyme elevations were relatively frequent; 76.7% of patients showed elevated ALT levels, and 74.4% had elevated AST levels, with grade 3 or higher elevations present in a notable proportion (ALT: 9.3%; AST: 16.3%).

Electrolyte imbalances were also prevalent. Hyponatremia affected over 80% of patients, predominantly grade 1–2, with only one case of grade 3. Hypocalcemia was observed in 55.8% of patients, most commonly at grade 1 severity. Additionally, hypoalbuminemia was frequent (93.0%), and over half of the cohort experienced grade 2 hypoalbuminemia (Table 4).

## 4. Discussion

In this retrospective study involving patients with relapsed or refractory germ cell tumors undergoing HDCT/ASCT, we investigated the relationship between pre-transplant clinical, laboratory, and morphometric parameters and hematopoietic recovery kinetics. Our results indicate that age and post-transplant nadir hemoglobin levels were independently associated with prolonged hematologic engraftment, whereas classical body composition parameters, such as skeletal muscle and fat area, showed no significant correlation, with the exception of the VFA/SFA ratio.

We found that increasing age was significantly associated with a prolonged neutrophil engraftment time. This observation is in line with previous findings suggesting that hematopoietic recovery is affected by aging-related declines in bone marrow regenerative capacity. Specifically, Fedorov et al. demonstrated that patients over 75 years old experienced significantly longer WBC and platelet engraftment times compared to younger patients despite similar transplant-related mortality and hospitalization durations [20]. Similarly, Elçin Erdoğan Yücel et al. observed a statistically significant difference in WBC engraftment between multiple myeloma patients aged above and below 65 years, with median engraftment durations of 12 and 10 days, respectively [21]. These observations are in concordance with our current findings and support the consideration of age as a clinically relevant factor in post-transplant recovery trajectories.

Furthermore, our study revealed a significant association between lower post-transplant hemoglobin nadirs and prolonged neutrophil engraftment. Despite erythrocyte transfusions being administered during the post-transplant period, patients with lower nadir hemoglobin values exhibited delayed hematologic recovery. This suggests that not only symptomatic anemia but also optimal hemoglobin management may play a role in facilitating timely engraftment. As patients undergoing HSCT frequently require transfusional support until red blood cell and platelet engraftment is complete, a better understanding of transfusion needs may help to minimize complications due to overtransfusion. The American Association of Blood Banks recommends transfusion in asymptomatic patients when hemoglobin levels fall below 7–8 g/dL [22]. Supporting this, Tabasi et al. reported that lower pre-transplant hemoglobin levels were associated with increased post-transplant transfusion requirements, highlighting the impact of pre-transplant hematologic status on recovery trajectory [23]. Although their study focused on pre-transplant hemoglobin, our findings expand this perspective by highlighting the predictive significance of post-transplant hemoglobin nadirs on engraftment kinetics.

Consistent with prior reports, the association between older age and prolonged engraftment likely reflects diminished hematopoietic reserve, whereas a lower hemoglobin may serve as a surrogate for greater treatment-related toxicity or bleeding—both plausibly contributing to delayed hematologic recovery.

Interestingly, although the SMI, PMI, and TFA were not significantly associated with engraftment duration in our cohort, a significant positive correlation between the VFA/SFA ratio and prolonged neutrophil recovery was observed. It should not be overlooked that the lack of statistical significance observed for the SMI, PMI, and TFA may be at least partly attributable to the limited sample size. This observation aligns with the growing body of evidence suggesting that altered fat distribution—particularly increased visceral adiposity—may influence transplant-related outcomes. Notably, while sarcopenia and frailty have traditionally been linked to adverse post-transplant recovery trajectories, our findings highlight that visceral fat predominance, possibly through systemic inflammatory pathways or dysregulated metabolic signaling, may also play a detrimental role in delaying hematologic engraftment. It should be noted that unmeasured factors such as general physical fitness or cytokine profiles may also complicate this effect. In this context, the study by M. Pamukçuoğlu et al., which evaluated frailty in 98 patients (51 of whom underwent autologous transplantation), demonstrated that the neutrophil engraftment time was significantly prolonged in frail patients compared to non-frail counterparts [24]. This finding supports the notion that sarcopenia and frailty may impair hematologic recovery, potentially through systemic inflammation, reduced physiological reserve, and altered bone marrow niche signaling. Taken together, our results reinforce the prognostic relevance of chronological age, visceral adiposity, and sarcopenia-related frailty in influencing post-transplant recovery trajectories. Therefore, integrating body composition profiling—encompassing both muscle and fat metrics—into the pre-transplant assessment may enhance risk stratification and personalized supportive strategies in patients undergoing HDCT-ASCT.

Moreover, we observed strong associations between muscle indices and several biochemical markers. The SMI was positively correlated with serum albumin and calcium levels and inversely correlated with liver transaminases (ALT and AST), reflecting a more favorable nutritional and metabolic profile. Similarly, the PMI was positively associated with the platelet count. These correlations reinforce the concept that muscle mass may serve as a surrogate for physiological reserve and systemic homeostasis.

Our findings extend and strengthen the existing literature in several ways. First, they derive from an understudied population—relapsed/refractory GCT patients undergoing HDCT/ASCT—where evidence on body composition and engraftment is scarce. Second, by evaluating a comprehensive panel of L3 CT-derived metrics (SMI, PMI, SFA, VFA, TFA, and VFA/SFA) within the same cohort and adjusting for key covariates (age, hemoglobin, weight, and BSA), we show that visceral fat predominance (VFA/SFA), rather than classical muscle or total fat areas, is related to delayed neutrophil recovery. Third, we corroborate age as a clinically meaningful correlate of engraftment and add novel evidence that lower post-transplant hemoglobin nadirs are independently associated with slower hematologic recovery, complementing prior work that focused on pre-transplant hemoglobin. Finally, by linking muscle indices with nutritional/metabolic markers (albumin, calcium, and transaminases) and conducting analyses under standardized supportive-care protocols, we provide biologically coherent and clinically actionable signals that can inform pre-transplant risk stratification and targeted optimization (e.g., metabolic and anemia management) in patients considered for HDCT/ASCT.

## 5. Conclusions

In conclusion, our study highlights the prognostic significance of both clinical and morphometric parameters in influencing hematopoietic recovery following HDCT-ASCT in patients with relapsed or refractory germ cell tumors. Increasing age and lower post-transplant nadir hemoglobin levels were independently associated with prolonged neutrophil engraftment, underlining the relevance of biological aging and hematologic reserve in post-transplant kinetics. While classical muscle and fat area indices did not predict engraftment duration, the visceral-to-subcutaneous fat area ratio emerged as a potential morphological marker that was associated with delayed recovery. These findings suggest that fat distribution, particularly visceral adiposity, alongside sarcopenia-related frailty, may affect hematologic regeneration, possibly through systemic inflammation or bone marrow niche disruption.

The strong correlation observed between muscle indices and biochemical parameters such as albumin, calcium, transaminases, and platelet count further supports the utility of body composition profiling as a surrogate for physiological resilience. The integration of comprehensive clinical, laboratory, and morphometric assessments into the pre-transplant evaluation may therefore enhance risk stratification and enable the development of individualized supportive care strategies aimed at optimizing outcomes in patients undergoing HDCT-ASCT.

## 6. Limitations

This retrospective, single-center study with a small cohort (*n* = 43) presents limited generalizability and statistical power, raising the risk of overfitting and false negatives (e.g., for SMI/PMI/TFA). Although salvage therapy was predominantly CE with standardized supportive care, residual heterogeneity (e.g., G-CSF timing and antimicrobial/transfusion practices) may confound the associations. Only pre-transplant CTs were analyzed, but variable imaging–ASCT intervals and incomplete historical imaging (selection bias) are possible. The multivariable adjustment was limited (no objective fitness/frailty or cytokine data), so residual confounding cannot be excluded; the association with post-transplant hemoglobin should be viewed as associative rather than causal. External validation in larger, and multicenter prospective cohorts is warranted.

## 7. Highlights

**Figure 4:**
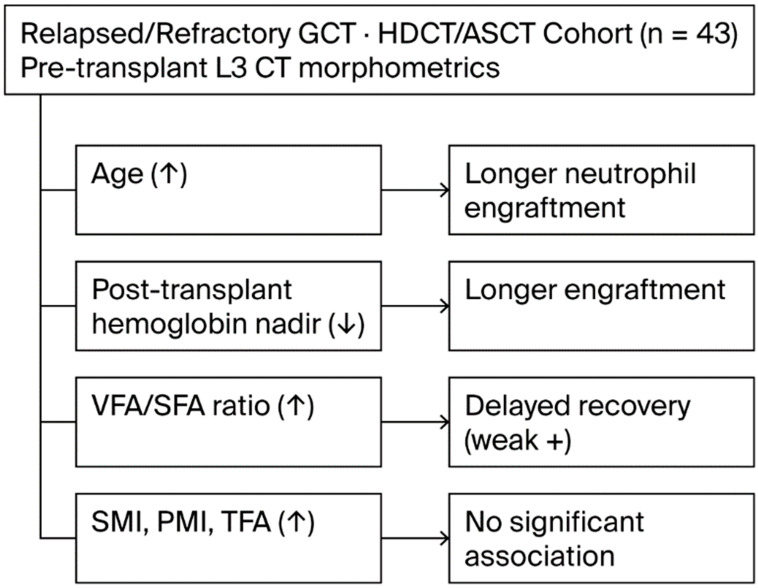
Summary of clinical and morphometric correlates of engraftment after HDCT/ASCT.

Older age and lower post-transplant hemoglobin are independently associated with longer engraftment after HDCT/ASCT. Among the morphometrics, the VFA/SFA (visceral adiposity predominance) shows a weak positive association with delayed engraftment; the SMI, PMI, and TFA are not predictive. The results support pre-transplant risk stratification using simple clinical and imaging metrics (Figure 4).

## 8. Future Directions

Validation in prospective, multicenter cohorts with adequate power is warranted, using standardized conditioning/supportive-care protocols, harmonized imaging, and richer phenotyping (HU-based muscle quality, frailty/functional metrics, and cytokine–inflammation panels). Studies should extend follow-up to clinical outcomes (infection burden, readmissions, and PFS/OS) and test interventions—such as anemia/metabolic optimization and prehabilitation—for their effects on engraftment time. Finally, they should develop and externally validate predictive models that integrate clinical (age and hemoglobin), morphometric (VFA/SFA), and laboratory variables to guide individualized supportive care.

## Figures and Tables

**Table 1:** Patients’ characteristics.

Variable	Category	N	%
Primary Tumor Localization	Gonadal	43	100.0
Histology	Non-Seminomatous	40	93.0
	Seminomatous	3	7.0
Non-Seminomatous Subtypes	Mixed Germ Cell Tumor	32	74.4
	Choriocarcinoma	1	2.3
	Embryonal Carcinoma	2	4.7
	Yolk Sac Tumor	4	9.3
	Immature Teratoma	1	2.3
Stage At Diagnosis	Stage < 3	21	48.8
	Stage ≥ 3	22	51.2
IGCCCG Risk Classification	Good (Group 1)	12	27.9
	Intermediate (Group 2)	6	14.0
	Poor (Group 3)	25	58.1
Metastasis Sites	Lung	26	60.5
	Lymph Nodes	42	97.7
	Liver	9	20.9
	Bone	7	16.3
	Brain	3	7.0
Response to Pre-HDCT Therapy	CR or Marker-Negative PR	24	55.8
	Marker-Positive PR or SD	18	41.9
	PD	1	2.3
Timing of HDCT	After 2 Lines	34	79.1
	After 3 Lines	9	20.9
HDCT Regimen	CE	40	93.0
	ICE	3	7.0
Response to HDCT	CR or Marker-Negative PR	28	65.1
	SD or Marker-Positive PR	9	20.9
	PD	6	14.0

**Table 2:** Correlation of Pre-HDCT/ASCT body composition indices with laboratory parameters.

			Creatine	ALT	AST	Sodium	Calcium	Albumin	Platelet	Hemoglobin	Neutrophil
Spearman’s rho	PRE-HDCT/ASCT SMI	Correlation Coefficient	−0.125	−0.516	−0.295	0.311	0.456	0.414	0.186	0.253	0.350
		Sig. (1-tailed)	0.212	0.000	0.028	0.021	0.001	0.003	0.116	0.051	0.011
		*n*	43	43	43	43	43	43	43	43	43
	PRE-HDCT/ASCT PMI	Correlation Coefficient	0.010	−0.385	−0.239	0.239	0.234	0.217	0.353	0.120	0.027
		Sig. (1-tailed)	0.473	0.005	0.061	0.061	0.065	0.082	0.010	0.222	0.432
		*n*	43	43	43	43	43	43	43	43	43
	PRE-HDCT/ASCT TFA	Correlation Coefficient	0.214	−0.231	−0.150	0.215	0.083	−0.009	0.100	0.115	0.175
		Sig. (1-tailed)	0.084	0.068	0.169	0.083	0.297	0.477	0.261	0.232	0.130
		*n*	43	43	43	43	43	43	43	43	43
	PRE-HDCT/ASCT VFA/SFA	Correlation Coefficient	−0.138	−0.049	.004	−0.149	−0.179	−0.106	0.058	0.053	0.153
		Sig. (1-tailed)	0.189	0.377	0.489	0.171	0.126	0.250	0.357	0.367	0.164
		*n*	43	43	43	43	43	43	43	43	43

**Table 3:** Correlation between pre-HDCT/ASCT body composition parameters and engraftment duration.

			PRE HDCT/ASCT SMI	PRE HDCT/ASCT PMI	PRE HDCT/ASCT TFA	PRE HDCT/ASCT VFA/SFA
Spearman’s rho	Engraftment Duration	Correlation Coefficient	0.050	0.080	0.113	0.297 *
		Sig. (1-tailed)	0.375	0.305	0.235	0.027
		*n*	43	43	43	43

* VFA/SFA ratio exhibited a statistically significant correlation with engraftment duration.

**Table 4:** Treatment-related hematologic and biochemical toxicities.

Toxicity Type	Grade	Frequency (*n*)	Percentage (%)
Neutropenia	Grade 1 (2000–1500/mm^3^)	0	0.0%
	Grade 2 (1500–1000/mm^3^)	0	0.0%
	Grade 3 (1000–500/mm^3^)	0	0.0%
	Grade 4 (<500/mm^3^)	43	100.0%
Anemia	Grade 1 (Hemoglobin 12–10 g/dL)	10	23.3%
	Grade 2 (10–8 g/dL)	30	69.8%
	Grade 3 (<8 g/dL)	3	7.0%
	Grade 4 (Life-threatening)	0	0.0%
Thrombocytopenia	Grade 1 (150,000–75,000/mm^3^)	0	0.0%
	Grade 2 (75,000–50,000/mm^3^)	0	0.0%
	Grade 3 (50,000–25,000/mm^3^)	0	0.0%
	Grade 4 (<25,000/mm^3^)	43	100.0%
Creatinine Elevation	Normal	31	72.1%
	Grade 1 (up to 1.5× baseline)	7	16.3%
	Grade 2 (1.5–3× baseline)	3	7.0%
	Grade 3 (3–6× baseline)	2	4.7%
	Grade 4 (>6× baseline)	0	0.0%
AST Elevation	Normal	11	25.6%
	Grade 1 (≤3× ULN)	15	34.9%
	Grade 2 (3–5× ULN)	10	23.3%
	Grade 3 (5–20× ULN)	6	14.0%
	Grade 4 (>20× ULN)	1	2.3%
ALT Elevation	Normal	10	23.3%
	Grade 1 (≤3× ULN)	18	41.9%
	Grade 2 (3–5× ULN)	11	25.6%
	Grade 3 (5–20× ULN)	3	7.0%
	Grade 4 (>20× ULN)	1	2.3%
Hyponatremia	Normal	7	16.3%
	Grade 1 (130–135 mmol/L)	22	51.2%
	Grade 2 (125–129 mmol/L)	13	30.2%
	Grade 3 (120–124 mmol/L)	1	2.3%
	Grade 4 (<120 mmol/L)	0	0.0%
Hypocalcemia	Normal	19	44.2%
	Grade 1 (corrected Ca < 8 mg/dL)	20	46.5%
	Grade 2 (7–8 mg/dL)	4	9.3%
	Grade 3 (6–7 mg/dL)	0	0.0%
	Grade 4 (<6 mg/dL)	0	0.0%
Hypoalbuminemia	Normal	3	7.0%
	Grade 1 (3–3.4 g/dL)	14	32.6%
	Grade 2 (2–3 g/dL)	23	53.5%
	Grade 3 (<2 g/dL)	3	7.0%
	Grade 4 (Life-threatening)	0	0.0%

## Data Availability

This manuscript does not report data generation or analysis. Therefore, there are no datasets available for public access.

## Abbreviations

AFP	Alpha-fetoprotein
ANC	Absolute neutrophil count
AST	Aspartate aminotransferase
CDCT	Conventional-dose chemotherapy
CT	Computed tomography
Hb	Hemoglobin
HU	Hounsfield unit
IHC	Immunohistochemistry
OS	Overall survival
PFS	Progression-free survival
PR	Partial response
SALL4	Spalt-like transcription factor 4
SFA	Subcutaneous fat area (cm^2^)
TAMA	Total abdominal muscle area (cm^2^)
VFA	Visceral fat area (cm^2^)
ALT	Alanine aminotransferase
ASCT	Autologous stem cell transplantation
BMI	Body mass index
CE	Carboplatin–etoposide
GCT	Germ cell tumor
HDCT	High-dose chemotherapy
ICE	Ifosfamide–carboplatin–etoposide
L3	Third lumbar vertebral level
PD	Progressive disease
PMI	Psoas muscle index (cm^2^/m^2^)
R^2^	Coefficient of determination
SD	Stable disease
SMI	Skeletal muscle index at L3 (cm^2^/m^2^)
TFA	Total fat area (cm^2^)
VFA/SFA	Visceral-to-subcutaneous fat area ratio
